# Effect of eribulin on patients with metastatic breast cancer: multicenter retrospective observational study in Taiwan

**DOI:** 10.1007/s10549-018-4778-y

**Published:** 2018-04-05

**Authors:** Kun-Ming Rau, Fu Ou-Yang, Ta-Chung Chao, Yao-Lung Kuo, Tsui-Fen Cheng, Tsu-Yi Chao, Dar-Ren Chen, Yen-Dun Tzeng, Being-Whey Wang, Chun-Yu Liu, Ming-Hung Hu, Yin-Che Lu, Wei-Jen Ou, Chin-Ho Kuo, Chieh-Han Chuang, Jung-Yu Kan, Fang-Ming Chen, Ming-Feng Hou

**Affiliations:** 1 Division of Hematology-Oncology, Kaohsiung Chang Gung Memorial Hospital, No. 123, Dapi Rd, Niaosong Dist., Kaohsiung, 833 Taiwan (ROC); 2 Division of Breast Surgery, Department of Surgery, Kaohsiung Medical University Hospital, No. 100, Tzyou 1st Road, Kaohsiung, 807 Taiwan (ROC); 3 Division of Medical Oncology, Taipei Veterans General Hospital, No. 201, Sec. 2, Shipai Rd, Beitou District, Taipei, 11217 Taiwan (ROC); 4 Department of Surgery, National Cheng-Kung University Hospital, No. 138, Sheng Li Road, Tainan, 704 Taiwan (ROC); 5 Breast Cancer Center, Shin Kong Wu Ho-Su Memorial Hospital, B3, No. 135, Wenchang Rd., Shilin Dist., Taipei, 111 Taiwan (ROC); 6 Division of Hematology-Oncology, Taipei Medical University- Shuang Ho Hospital, No. 291, Zhongzheng Rd., Zhonghe District, New Taipei City, 23561 Taiwan (ROC); 7 Department of Surgery, Changhua Christian Hospital, No. 135, Nanxiao St., Changhua, 500 Taiwan (ROC); 8 Division of General Surgery, Kaohsiung Veterans General Hospital, No. 386, Dazhong 1st Rd., Zuoying Dist., Kaohsiung, 813 Taiwan (ROC); 9 Division of Hematology and Oncology, Cardinal Tien Hospital, No. 362, Zhongzheng Rd., Xindian Dist., New Taipei City, 231 Taiwan (ROC); 10 Division of Hematology-Oncology, Chia-Yi Christian Hospital, No. 539, Zhongxiao Rd., East Dist., Chiayi, 60002 Taiwan (ROC); 11 Division of Medical Oncology, Landseed Hospital, No. 77, Guangtai Rd., Pingzhen Dist., Taoyuan, 324 Taiwan (ROC); 12 Kaohsiung Breast Cancer Prevention and Education Society, 10B, No. 100, Tzyou 1st Road, Kaohsiung, 807 Taiwan (ROC); 13 Department of Surgery, Kaohsiung Municipal Ta-Tung Hospital, No. 68, Zhonghua 3rd Rd., Qianjin Dist., Kaohsiung, 801 Taiwan (ROC); 14 Cancer Center, Kaohsiung Municipal Ta-Tung Hospital, No. 68, Zhonghua 3rd Rd., Qianjin Dist., Kaohsiung, 801 Taiwan (ROC); 15 Department of Surgery, Faculty of Medicine, College of Medicine, Kaohsiung Medical University, No. 100, Tzyou 1st Road, Kaohsiung, 807 Taiwan (ROC); 16 Department of Surgery, Kaohsiung Municipal Hsiao-Gang Hospital, No. 482, Shanming Rd, Siaogang Dist., Kaohsiung, 812 Taiwan (ROC)

**Keywords:** Eribulin, Metastatic breast cancer, Efficacy, Safety, Real world, Taiwanese women

## Abstract

**Purpose:**

The aim of this study was to confirm the therapeutic role of eribulin on Taiwanese women with metastatic breast cancer.

**Methods:**

This retrospective study examined 449 females who received eribulin between March 2014 and June 2017 at 14 hospitals in Taiwan for treatment of locally advanced or metastatic breast cancer.

**Results:**

The survival rate at 24 months was 57.2% (95% CI 51.0–62.9%) and the median time to treatment failure (TTF) was 3.91 months (95% CI 3.45–3.94). A total of 175 patients (40.1%) received eribulin for fewer than 90 days and the others received it for 90 days or more. Eight patients (1.83%) had complete remission, 82 (18.8%) had partial remission, 202 (46.3%) had stable disease, and 144 (33.0%) had progressive disease (PD). Patients’ tumors with the luminal A subtype had a significantly better objective response rate. Kaplan–Meier analysis indicated that hormone receptor positivity, luminal A subtype, receipt of eribulin as the 1st to 3rd line therapy, and metastasis to fewer than 4 organs were significantly associated with longer TTF. Stepwise multivariate analysis showed that only receipt of eribulin as the 1st to 3rd line therapy was significantly associated with TTF (HR 1.49, *p* < 0.001). All toxicities were manageable and only 18 patients (4.1%) discontinued treatment due to adverse events.

**Conclusions:**

Eribulin appears to have better efficacy and cause fewer adverse events, especially neutropenia, in Taiwanese women than Western women.

## Introduction

Breast cancer is the most common cancer among women worldwide, and approximately 10% of newly diagnosed cases are stage IV (metastatic) [1]. Several treatments are available for patients with metastatic breast cancer (MBC), but none of them are curative treatments, so these patients tend to have poor long-term survival rates, with median survival times of 18–24 months [2]. The goals of most treatments of these patients are palliation and improvement in the quality of life. Taxanes and anthracyclines are the standard adjuvant and first-line treatments for women with MBC, but these treatments are not always successful due to the development of drug resistance [3]. Thus, it is difficult to treat patients after failure of these therapies, and there are no established regimens for subsequent treatment.

Microtubules have an important function in the mitosis of normal and cancer cells. Thus, several agents that target microtubules are used to treat different cancers. These include paclitaxel and docetaxel, which prevent microtubule depolymerization, and vincristine and vinblastine, which inhibit microtubule formation [4]. These drugs disrupt normal function of microtubule during mitosis, leading to cell death.

Eribulin (MW: 826.0, C_40_H_59_NO_11_. CH_4_O_3_S) is an anti-tubulin agent that was first isolated from a marine sponge [5]. Studies of its mechanism of action indicated that, in contrast to other microtubule-targeting agents, eribulin prevents the formation of cross-links between different sulfhydryl groups in β-tubulin [6]. Thus, eribulin inhibits microtubule growth, leading to an accumulation of non-functional aggregates of tubulin, and then mitosis arrests at metaphase/anaphase. In addition, studies of several lines of cancer cells indicated that eribulin has additive or synergistic effects with other antineoplastic agents [6].

Eribulin was initially approved in 2010 for treatment of MBC in patients who previously completed at least 2 chemotherapy regimens [7]. Although it is now approved for this indication in many countries, there are limited studies of its effect in Asian women. There was a study of the efficacy of eribulin in 80 Japanese women with MBC [8], but most previous studies only included ~ 2% Asian women [9–12]. Several clinical trials had proved the effect and safety of eribulin on locally advanced breast cancer (LABC) or MBC [6], but the real-world experiences of eribulin treatment were still few to be reported, especially for patients who do not fit the criteria of clinical trials. The benefits and toxicities of eribulin may differ among Western and Asian women. Here we performed a multicenter, retrospective investigation to determine the effect of eribulin in Taiwanese patients with MBC. Because some doctors would choose either capecitabine or vinorelbine as a salvage or maintenance therapy for advanced breast cancer after anthracycline or taxane, here we also would check the impact of previous exposure to these two drugs on eribulin treatment.

## Methods

### Patients

The clinical data of patients who received eribulin between March 2014 and June 2017 at 14 hospitals in Taiwan were retrospectively collected and reviewed (Clinical Trial Registry Number: NCT03245112). Included patients should be with pathological confirmed LABC or MBC, and had been treated by an anthracycline and a taxane regimen before, either as the adjuvant or metastatic purpose. There were no exclusions based on age or menopausal status, and none of the patients should be pregnant or nursing. The characteristics of tumors such as estrogen receptor (ER), progesterone receptor (PgR), human epidermal growth factor receptor 2 (Her-2), Ki-67, and molecular subtypes (luminal A, luminal B, Her-2 enriched, and triple-negative breast cancer [TNBC]) were recorded. Metastatic sites including lymph nodes and distant organs were also recorded and analyzed.

### Treatment

The treatment consisted of monotherapy with intravenous eribulin (over 2–5 min), which was administered at a dose of 1.4 mg/m^2^ on days 1 and 8 of a 21-day cycle. The reported data included characteristics of tumors, clinical parameters (e.g., site of metastases), treatment events (e.g., number of therapeutic cycles, start/end dates, and rationale for discontinuation), clinical response, use of supportive care medications (e.g., granulocytic colony stimulating factor), dose adjustment, and adverse events. The reported results are based on effectiveness analysis of data collected by June 2017.

### Outcome measures

The primary outcome measure is disease control rate (DCR), which was defined as proportions of patients who achieved complete response (CR), partial response (PR), and stable disease (SD) as the best response. Thus, the percentage of patients with LABC or MBC who achieved CR, PR, and SD were recorded during eribulin treatment. The secondary outcome measure is the safety of eribulin, i.e., the number of patients with adverse events (AE) and severity of AE which were assessed using the Common Terminology Criteria for Adverse Events (CTCAE), version 4.0. This includes all events that were not present before the initial administration of eribulin, pre-existing events that became more intense or more frequent, and events that were present upon initial eribulin administration, but became more severe following administration.

### Statistical analysis

Characteristics of patients and tumors, treatment duration, tumor response, and other categorical variables are summarized *n* (%), and age as median (range). The overall survival (OS) time was defined as the time from treatment onset to death or the last follow-up, which will be presented as mean with 95% confidence interval (CI). The time to treatment failure (TTF) was defined as the period from the first dose of eribulin to cancelation for any reason (including death, disease aggravation, treatment toxicity, or patient’s request), or was censored at the date of last follow-up for surviving patients remaining on treatment which will be presented as mean with 95% CI. Univariate analysis was performed to determine the association of tumor responses (objective response rate [ORR], CR + PR; and DCR, CR + PR + SD) with patient characteristics. The differences of tumor responses were compared using one-way ANOVA for age, and the Pearson Chi-square (*χ*^2^) test or Fisher’s exact test for other categorical variables. Univariate and multivariate binary logistic regression were also applied to determine the association between tumor responses and patient characteristics and presented as odds ratios (ORs), 95% CIs, and *p* values. Kaplan–Meier (K–M) curves for overall survival (OS) and TTF were also determined. Furthermore, the K–M curve and log-rank test were used to assess the association of TTF with different characteristics. In particular, the median, 95% CI, and *p* value of TTF for each characteristic were calculated using the log-rank test. Univariable Cox-regression analyses were also used to identify the association of TTF and different characteristics, and a stepwise multivariable Cox-regression analysis was used to analyze variables with *p* values below 0.2 in the univariable analysis. All statistical assessments were two-tailed and a *p* value below 0.05 was considered significant. All data analysis used Stata Statistical Software (Release 11, StataCorp LP, College Station, TX).

## Results

### Characteristics

The medical records of a total of 449 patients from 14 hospitals who received eribulin were retrospectively reviewed. Six patients were excluded because of loss to follow-up. Thus, we initially recruited 443 patients who were scheduled for treatment. Among these patients, 436 patients received at least one dose of eribulin and were used to assess efficacy, and 440 patients were used to assess safety.

The median age was 51.6 years old (range 22.2–81.0), 27.8% of patients were Her-2 positive, 64.9% were ER positive, and 49.1% were PgR positive. Analysis of the molecular subtypes indicated that 222 (50.9%) patients were luminal A, 68 (15.6%) were luminal B, 51 (11.7%) were Her-2 enriched, and 75 (17.2%) were TNBC. The median number of previous chemotherapy regimen was 3 (0–12). A total of 207 patients (47.5%) received eribulin as a 4th line or later treatment, 215 patients (49.3%) received eribulin as a 1st to 3rd line treatment, and the line of treatment was unknown for 14 patients (3.21%). There were 221 patients (50.7%) who received previous capecitabine and 217 patients (47.8%) who received previous vinorelbine; 142 (32.6%) of patients received neither of these agents, and 144 (33.0%) of patients received both agents. Most patients had metastasis to a single site (*n* = 224, 51.4%); bone (*n* = 157, 36.0%), lung (*n* = 141, 32.3%), and liver (*n* = 118, 27.1%) were the most common sites of metastases. (Table 1).

**Table 1:** Clinical characteristics of enrolled patients. (*n* = 436)

Characteristic	*n*	%
ER		
Positive	283	64.91
Negative	139	31.88
Unknown	14	3.21
PR		
Positive	214	49.08
Negative	207	47.48
Unknown	15	3.44
Her-2		
Positive	121	27.75
Negative	300	68.81
Unknown	15	3.44
Molecular subtype		
Luminal A	222	50.92
Luminal B	68	15.60
Her-2 enriched	51	11.70
TNBC	75	17.20
Unknown	20	4.59
Previous chemotherapy		
Capecitabine	221	50.69
Vinorelbine	217	49.77
Capecitabine alone	77	17.66
Vinorelbine alone	73	16.74
Capecitabine & Vinorelbine naïve	142	32.57
Capecitabine & Vinorelbine	144	33.03
Therapy line of eribulin		
1	59	13.53
2	67	15.37
3	89	20.41
> 3	207	47.48
Unknown	14	3.21
Sites with metastasis		
1	224	51.38
2	84	19.27
3	75	17.20
≥ 4	53	12.16
Organs with metastasis		
Bone	157	36.01
Lung	141	32.34
LN	81	18.58
Liver	118	27.06
Brain	53	12.16
Skin	28	6.42

*ER* estrogen receptor, *Her2* human epidermal growth factor receptor 2, *PR* progesterone receptor, *LN* lymph node, *TNBC* triple-negative breast cancer

### Efficacy of treatment

**Fig. 1:**
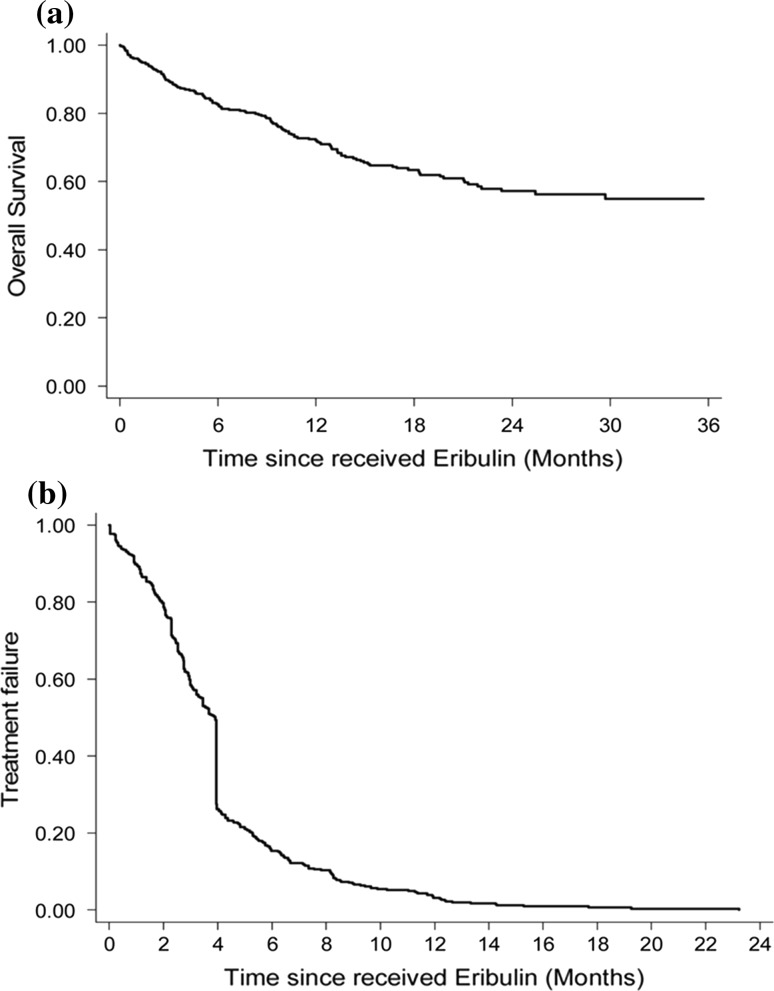
Kaplan–Meier curves of **a** overall survival and **b** time to treatment failure. The mean OS was 13.4 months (95% CI 0.26–35.06) and the median TTF was 3.91 months (95% CI 3.45–3.94 months). Abbreviations: *OS* overall survival, *TTF* time to treatment failure. *CI* confidence interval, *NA* not assessed

As of Nov. 30, 2017, 142 patients (32.6%) had expired, so the median OS time could not be calculated. The OS rate at 24 months was 57.2% (95% CI 51.0–62.9%, Fig. 1a) and the median TTF was 3.91 months (95% CI 3.45–3.94, Fig. 1b). Stratify the patients by the treatment duration, the results indicated that 175 patients (40.1%) received eribulin for fewer than 90 days and the others received eribulin for 90 days or more. The median cycles of eribulin administration were 4.

Analysis of tumor response indicated that 8 patients (1.83%) were CR, 82 (18.8%) were PR, 202 (46.3%) were SD, and 144 (33.0%) were PD (Table 2). The ORR was 20.6% and the DCR was 67.0%. The primary reasons for treatment discontinuation were disease progression (88.6%), adverse events (4.1%), patient’s request (3.7%), and death (1.9%) (data not shown).

**Table 2:** Treatment and tumor responses

Characteristic	*n* (%) or as indicated	
Eribulin treatment duration		
< 90 days	175	(40.14%)
≥ 90 days	261	(59.86%)
TTF, months		
Mean ± SD	3.99	(± 3.06)
Median (range)	3.91	(0.03–23.23)
Response		
CR	8	(1.83%)
PR	82	(18.81%)
SD	202	(46.33%)
PD	144	(33.03%)

*CR* complete response, *PD* progressive disease, *PR* partial response, *SD* stable disease

Different subtypes also had different responses to eribulin. In general, luminal A subtype had significantly better ORR (25.7%) than those with the luminal B (17.7%), Her-2 enriched (21.6%), TNBC (9.33%), and unknown (15.0%) subtypes (*p* = 0.032). Moreover, univariate and multivariate analysis of factors associated with ORR indicated that patients with the TNBC subtype had a lower ORR than those with luminal A subtype (OR 0.30, 95% CI 0.13–0.69, *p* = 0.004), this association remained in the multivariable analysis (OR 0.18, 95% CI 0.04–0.77, *p* = 0.021) (data not shown). We also show that ORR was higher in patients who were prior capecitabine and vinorelbine naïve (25.4%), received eribulin as the first line (28.8%) or second to third line (21.2%), and had metastasis to fewer than 4 sites (88.9%), although none of these differences were statistically significant (Table 3).

**Table 3:** Relationship of tumor characteristics with response

Characteristics	CR + PR		SD		PD		*P*
	*n*	%	*n*	%	*n*	%	
Total cases	90	20.64	202	46.33	144	33.03	
Molecular subtype							0.032^b^
Luminal A	57	25.68	98	44.14	67	30.18	
Luminal B	12	17.65	37	54.41	19	27.94	
Her-2 enriched	11	21.57	21	41.18	19	37.25	
TNBC	7	9.33	34	45.33	34	45.33	
Unknown	3	15.00	12	60.00	5	25.00	
Previous chemotherapy regimen							
Capecitabine	41	18.55	102	46.15	78	35.29	0.439^b^
Vinorelbine	40	18.43	98	45.16	79	36.41	0.267^b^
Capecitabine alone	14	18.18	35	45.45	28	36.36	0.406^b^
Vinorelbine alone	13	17.81	31	42.47	29	39.73	
Capecitabine & Vinorelbine naïve	36	25.35	69	48.59	37	26.06	
Capecitabine & Vinorelbine	27	18.75	67	46.53	50	34.72	
Eribulin therapy line after metastases							0.210^b^
1	17	28.81	29	49.15	13	22.03	
2–3	33	21.15	65	41.67	58	37.18	
> 3	39	18.84	96	46.38	72	34.78	
Unknown	1	7.14	12	85.71	1	7.14	
Metastatic sites							0.392^b^
< 4	80	88.89	181	89.60	122	84.72	
≧ 4	10	11.11	21	10.40	22	15.28	

^a^*p* value estimated by one-way ANOVA test

^b^*p* value estimated by one-way Chi-squared test

**Fig. 2:**
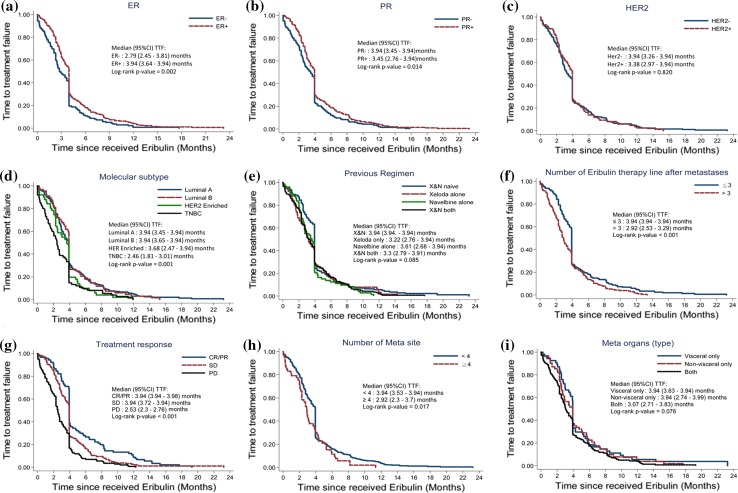
Kaplan–Meier curves of time to treatment failure (TTF) according to **a** ER status, **b** PR status, **c** Her-2 status, **d** molecular subtype, **e** previous regimen, **f** therapy line of eribulin, **g** treatment response, **h** number of metastatic sites, **i** metastatic organs. Medians and 95% confidence intervals (95% CIs) were calculated using the log-rank test

We used K–M survival analysis and the log-rank test to assess the relationship of different demographic and clinical characteristics with TTF. These results show that luminal A subtype, receipt of eribulin as the 1st, 2nd, or 3rd line, ORR, and metastasis to fewer than four organs were significantly associated with longer TTF (*p* < 0.05 for all comparisons) (Fig. 2).

We also performed univariate and multivariate analysis of the relationship of TTF with different demographic and clinical characteristics. The univariate results showed that TNBC subtype (HR 1.65, *p* < 0.001), previous treatment with capecitabine and vinorelbine (HR 1.28, *p* = 0.042), and metastasis to 4 sites or more (HR 1.40, *p* = 0.024) were significantly associated with worse TTF. The stepwise multivariate analysis, which considered variables whose *p* values were less than 0.2 in the univariate analysis, showed that only receipt of eribulin as the 1st, 2nd, or 3rd line therapy was significantly associated with better TTF (HR 1.49, *p* < 0.001) (Table 4).

**Table 4:** Univariate and multivariate Cox proportional hazard regression analysis of factors associated with time to treatment failure (TTF). (*n* = 436)

Variables	Univariate			Multivariate^a^			Multivariate^b^		
	HR	95%CI	*P*	HR	95%CI	*P*	HR	95%CI	*P*
Molecular subtype									
Luminal A	1.00			1.00			1.00		
Luminal B	0.99	0.76–1.31	0.965	1.08	0.6–1.93	0.796	0.95	0.72–1.26	0.729
Her-2 enriched	1.32	0.97–1.79	0.077	1.78	0.73–4.33	0.205	1.50	0.76–2.97	0.241
TNBC	1.65	1.26–2.14	< 0.001	1.92	0.96–3.83	0.066	1.79	0.93–3.47	0.083
Previous capecitabine	1.16	0.96–1.4	0.126	–			–		
Previous vinorelbine	1.20	0.99–1.45	0.061	–			–		
Previous regimen									
Capecitabine & vinorelbine naïve	1.00			1.00			1.00		
Capecitabine alone	1.25	0.95–1.66	0.115	1.00	0.73–1.37	0.994	1.11	0.82–1.5	0.504
Vinorelbine alone	1.33	1–1.77	0.052	1.06	0.76–1.47	0.721	1.04	0.76–1.41	0.821
Capecitabine & vinorelbine	1.28	1.01–1.61	0.042	0.94	0.7–1.26	0.663	1.01	0.77–1.32	0.960
Eribulin therapy line after metastases									
≦ 3	1.00			1.00			1.00		
> 3	1.25	0.95–1.66	0.115	1.47	1.16–1.86	< 0.001	1.49	1.19–1.86	**< 0.001**
Meta site no.									
< 4	1.00			1.00			1.00		
≧ 4	1.40	1.04–1.86	0.024	1.31	0.85–2.02	0.226	1.11	0.82–1.51	0.489
Organs with metastasis^c^				–			–		
Visceral only	Ref								
Non-visceral only	1.12	0.77–1.62	0.548						
Both	1.38	1–1.9	0.048						

1.00: Reference category

^a^Included all variables in univariate analysis

^b^Included variables with *p* value less than 0.2 from univariate analysis

^c^Unknown meta organ type was excluded (*n* = 176)

### Adverse events

We assessed the safety of eribulin by analysis of 440 patients, there were 18 patients (4.1%) who discontinued treatment due to AEs. Neutropenia (21.3%) and leukopenia (16.7%) were the major hematological adverse events. The most common non-hematological AEs were alopecia (26.4%), fatigue or lethargy (21.3%), and nausea (11.5%). A total of 86 patients (19.7%) received treatment with granulocyte colony stimulating factor for chemotherapy-induced neutropenia (Table 5).

**Table 5:** Adverse events. (*n* = 440)

	All grades	Grade 3	Grade 4	Use of G-CFS
Hematological				
Leukopenia	73 (16.7%)	8 (1.8%)	26 (6%)	
Neutropenia	93 (21.3%)	12 (2.8%)	41 (9.4%)	86 (19.7%)
Non-hematological				
Allergic reaction	21 (4.8%)	–	–	
Alopecia	115 (26.4%)	–	–	
Anorexia	25 (5.7%)	–	–	
Cough	7 (1.6%)	–	–	
Diarrhea	18 (4.1%)	–	–	
Fatigue/lethargy	93 (21.3%)	7 (1.6%)	–	
Fever	15 (3.4%)	–	–	
Hand-foot syndrome	16 (3.7%)	–	–	
Mucositis	41 (9.4%)	2 (1.0%)		
Peripheral neuropathy	41 (9.4%)	2 (0.5%)	–	
Vomiting	39 (8.9%)	1 (0.2%)	–	
Nausea	50 (11.5%)	–	–	
Constipation	4 (0.9%)	–	–	
Rash acneiform/skin rash	18 (4.1%)	–	–	

## Discussion

Several clinical studies of eribulin evaluating its safety and efficacy in real-world clinical settings had been published previously. For example, in Watanabe et al. [13], they reported a post-marketing observational study in Japanese patients with locally advanced or metastatic breast cancer, of these, 671 patients were included in the effectiveness analysis. They found that CR and PR were observed in 1.3 and 15.2% of patients, respectively. The ORR was 16.5%, DCR was 50.1%, clinical benefit rate was 22.4%, and the median TTF was 127 days. On the other hand, Garrone et al. performed a multicenter study of the effect of eribulin on 113 Italian women who were previously treated for MBC to assess its effect in an actual clinical setting [14]. They reported an ORR of 24%, and a clinical benefit rate of 35.4%. Moreover, after a median follow-up time of 29.6 months, the median PFS was 3.3 months, and the median OS was 11.6 months. They concluded that eribulin was safe and effective in a real-world clinical setting, in agreement with our results. Watanabe [15] also studied eribulin in a real-world clinical setting. In particular, he retrospectively examined the effect of eribulin monotherapy on survival of Japanese women with ER-positive and Her-2-negative MBC. Sixty-six women received eribulin and 227 received a conventional chemotherapy agent. The results indicated significantly better OS in patients receiving eribulin. Moreover, the survival benefit did not depend on which organs had metastases or the use of previous chemotherapy regimens. In agreement with the results of Watanabe [15], we found that all 7 of our patients who had CRs were Her-2 negative and ER positive, and 6 of them were PgR positive. Gamucci et al. [16] retrospectively studied 133 Italian women with advanced/metastatic breast cancer who previously received 2 or more lines of chemotherapy. They reported that the ORR was 21.1%, SD in 42.8% of patients, and the presence of PR or SD for at least 6 months in 38.3% of patients. They also found that eribulin was especially effective when given to women with Her-2-negative tumors.

There are several important results of the present retrospective study of the effect of eribulin on Taiwanese women with MBC or LABC. Similar to the results of a previous study of Japanese women [15], Taiwanese women who had hormone receptor-positive and Her-2-negative BC had better outcomes than those who were Her-2 positive. Although the pooled analysis of trial 301 and EMBRACE study reported that eribulin was more effective for TNBC, the researchers only compared it with the control arm [11]. Real-world data indicate that luminal A patients had better therapeutic outcomes than those who were Her-2 positive or with TNBC. In addition, TTF in the current study was consistent with the prior study [13]. In the current study, patients also had better overall survival time than reported in another study in a real other world setting [17]. This might be because the availability of more treatment options in recent years that extended the survival time of our patients.

There was greater variation in the number of lines on previous therapy before eribulin treatment for patients in the present study than in most previous studies. Our patient population was more like those in the EMBRACE study, and our results are at least non-inferior to those in the EMBRACE study [9]. In particular, we examined patients who received 1 prior regimen to more than 3 prior regimens (median: 4), we found that ORR was better for patients who received fewer prior treatments, and K-M analysis indicated a small increase in TTF of patients who received 3 or fewer lines of therapy, relative to those who received 4 or more lines (3.94 months vs. 2.92 months, *p* < 0.001, Fig. 2f), although DCR was not significantly different for those with different numbers of prior treatments. In addition, response rate in the current study was higher than that of previous phase II [18], phase IV [19], and retrospective studies [13] among Asian patient population. This suggests that eribulin could be considered for patients with a wide variety of treatment histories, and it is reasonable that earlier use of eribulin provides a better ORR.

Taiwan has provided reimbursement for use of eribulin by advanced breast cancer patients beginning in December 2014. Thus, prior to this date, patients received an anthracycline-based and taxane-based regimen. This is the reason we had so many patients who received eribulin, even after having received several prior treatments.

Compared with previous studies, we found that eribulin caused fewer and less severe AEs in Taiwanese women than in women treated in clinical trials [9, 10], but our AE results are comparable to the real-world experience reported by Iizumi et al. [17]. In particular, the rate of neutropenia was much lower in our population than reported for Westerners [9], and even for a Korean population [19] and for Japanese populations [8, 18]. Although further studies are needed to identify the molecular, genetic, environmental, and socioeconomic factors that could explain these differences, one of the reasons for the better efficacy and the fewer and less severe AEs in our patients may be that the national health insurance system of Taiwan requires certification of the quality of care in all hospitals. Thus, this policy may have contributed to the improved care and survival of our cancer patients [20]. Another reason for the fewer and less severe AEs in our patients may be that almost all patients were restricted to 2 mg as the highest dosage for every single injection, in an effort to control medical costs.

The major limitations of the present study are the retrospective and open label design, which could lead errors related to confounding or bias, and the use of a single treatment arm, with no comparators. Nonetheless, we examined a large population of Taiwanese women who received treatment of eribulin at 14 different hospitals in Taiwan, and used multivariable analysis to reduce the impact of confounding. The results of the present study of eribulin confirm that this drug is safe and effective when used to treat Taiwanese women with LABC or MBC who previously received at least 2 previous chemotherapy regimens that included an anthracycline and a taxane in either the adjuvant or the metastatic setting. Our data also confirm that eribulin maintains its favorable profile in terms of clinical effectiveness when used in daily clinical practice in heavily pretreated patients.
